# A bibliometric analysis of COVID-19 research in Africa

**DOI:** 10.1136/bmjgh-2021-005690

**Published:** 2021-05-10

**Authors:** Fatuma Hassan Guleid, Robinson Oyando, Evelyn Kabia, Audrey Mumbi, Samuel Akech, Edwine Barasa

**Affiliations:** 1 Policy Engagement & Knowledge Translation Unit, KEMRI-Wellcome Trust Research Programme, Nairobi, Kenya; 2 Health Economics Research Unit, KEMRI-Wellcome Trust Research Programme, Nairobi, Kenya; 3 Health Services Unit, KEMRI-Wellcome Trust Research Programme, Nairobi, Kenya; 4 Center for Tropical Medicine and Global Health, Nuffield Department of Medicine, University of Oxford, Oxford, UK

**Keywords:** COVID-19, public health, other study design

## Abstract

**Background:**

The COVID-19 pandemic has led to an unprecedented global research effort to build a body of knowledge that can inform mitigation strategies. We carried out a bibliometric analysis to describe the COVID-19 research output in Africa in terms of setting, study design, research themes and author affiliation.

**Methods:**

We searched for articles published between 1 December 2019 and 3 January 2021 from various databases including PubMed, African Journals Online, medRxiv, Collabovid, the WHO global research database and Google. All article types and study design were included.

**Results:**

A total of 1296 articles were retrieved. 46.6% were primary research articles, 48.6% were editorial-type articles while 4.6% were secondary research articles. 20.3% articles used the entire continent of Africa as their study setting while South Africa (15.4%) was the most common country-focused setting. The most common research topics include ‘country preparedness and response’ (24.9%) and ‘the direct and indirect health impacts of the pandemic’ (21.6%). However, only 1.0% of articles focus on therapeutics and vaccines. 90.3% of the articles had at least one African researcher as author, 78.5% had an African researcher as first author, while 63.5% had an African researcher as last author. The University of Cape Town leads with the greatest number of first and last authors. 13% of the articles were published in medRxiv and of the studies that declared funding, the Wellcome Trust was the top funding body.

**Conclusions:**

This study highlights Africa’s COVID-19 research and the continent’s existing capacity to carry out research that addresses local problems. However, more studies focused on vaccines and therapeutics are needed to inform local development. In addition, the uneven distribution of research productivity among African countries emphasises the need for increased investment where needed.

**Key questions**

**What is already known?**

- Africa’s contribution to global health research is low (1.3%) considering the high burden of infectious disease on the continent.

**What are the new findings?**

- Africa is contributing to the generation of COVID-19 knowledge by publishing primary and secondary research articles and editorial and commentary-type articles.
- African authors have made significant contributions to this productivity and are listed as first authors in 78.5% of the articles and last authors in 63.5% of the articles.
- South Africa has the highest COVID-19 research productivity among all the African countries.
- How countries prepared and responded was the most recurring research theme while studies on therapeutics and vaccines were under-represented.

**What do the new findings imply?**

- These findings highlight Africa’s capacity to carry out research relevant to its context.
- However, more effort is needed to ensure even distribution of research productivity across the continent.
- More clinical studies on vaccines and therapeutics are needed to provide evidence on efficacy and safety in African populations.

## Introduction

Since its emergence in China in late 2019,^1^ SARS-CoV-2, the virus that causes COVID-19, has infected over 83 million people and caused over 1.8 million deaths worldwide as of 5 January 2021.^2^ SARS-CoV-2 predominantly infects the airways, and disease can range from asymptomatic and mild respiratory infections to severe acute respiratory distress syndrome, with the latter resulting in organ failure in some individuals and eventually leading to death.^3–6^ In Africa, where the first case was reported in February 2020, there have been 2.8 million cases and over 68 000 deaths as of 6 January 2021.^7^ The pandemic, in addition to health system constraints and the burden of existing communicable diseases, has put considerable pressure on already weak health systems across the continent. In addition, measures put in place to control the spread of the virus^8^ have led to the closure of schools, businesses and social services which have generated significant setbacks to Africa’s heath programmes, economy and communities.^9–14^

As COVID-19 is an emerging infectious disease, the research community has responded rapidly to provide insight into how to control the pandemic and in the development of tests, therapeutics and vaccines. Research on COVID-19 has been predominantly from China, Europe and the USA,^15 16^ which is understandable as these regions have experienced more cases and deaths from the pandemic. For reasons that are still uncertain, Africa has experienced fewer cases and deaths in the initial phase of the COVID-19 pandemic compared with other continents. This suggests that local research that considers the local context is necessary to inform contextually relevant mitigation strategies and treatment options.

In this bibliometric analysis, we describe the research on COVID-19 that has been done in Africa in terms of the geographical spread of the research, the study methodologies used, the research trends, funding sources and contributions of authors from Africa to highlight local capacity for research and research areas that are neglected.

## Methods

### Data source

Published papers and grey literature were searched via a topic search (title/abstract) on the following databases: PubMed, African Journals Online, medRxiv, bioRxiv, Collabovid, the WHO global research database and Google for grey literature. Searches were restricted to those published between 1 December 2019 and 3 January 2021.

### Eligibility criteria and study selection

Only articles with a focus on COVID-19 in Africa were included. Four reviewers independently performed study selection and data extraction. There was no restriction on the type of articles that were included. However, only documents in the English language were considered for the analysis. Differences in opinion were settled by referral to a fifth review author.

### Search strategy

The key search words used were those listed for Africa plus keywords pertaining to COVID-19 or SARS-CoV-2 (full search terms are provided in the online supplemental file). These search words were used in the title/abstract fields. Retrieved articles were manually checked for validity of search strategy and articles that were outside the scope and any duplicates were removed.

10.1136/bmjgh-2021-005690.supp1 Supplementary data

### Analysis

Descriptive analyses were conducted to evaluate the characteristics and types of articles retrieved using Microsoft Excel. These included titles; author information including first author and last author, author countries and affiliations; journal source; funders and funder countries; the research objective; the study setting; and keywords. In order to depict relations among keywords, co-occurrence network analysis was done with VOSviewer (V.1.6.15), a software tool for constructing and visualising bibliometric networks. VOSviewer was also used to assess coauthorship among all the authors in the bibliography and an evaluation of how many of them were connected within documents authored or coauthored by individuals was conducted. The results for the network of actors analysis (supplementary figure 1) and keyword analysis (supplementary figure 2) are included included in the online supplemental file.

### Patient and public involvement

No patients were involved in this study.

## Results

### Publication output

**Figure 1:**
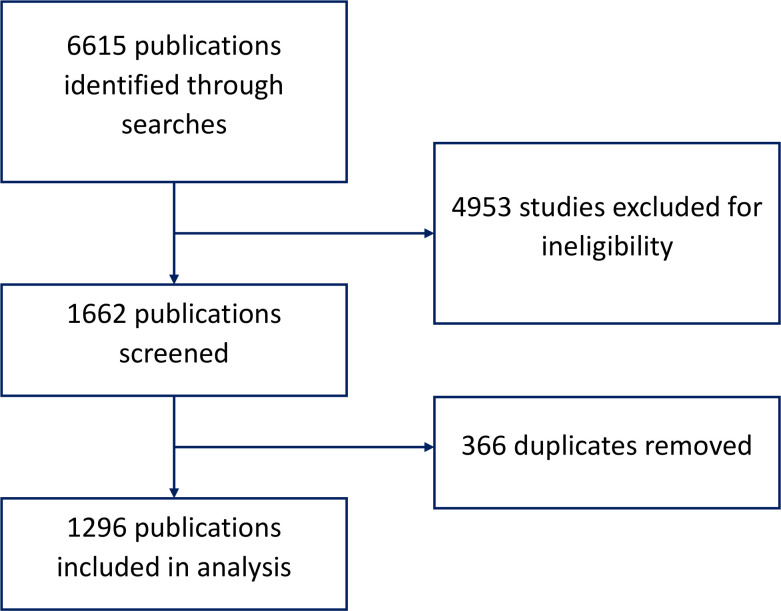
Flowchart for study selection.

The initial search yielded 6615 articles. After removing duplicates and articles that did not meet the eligibility criteria, we included 1296 articles in our analysis (figure 1).

Of the 1296 articles reviewed, 630 (48.6%) were non-original research articles (commentary, editorial, perspective pieces, etc), while 606 (46.6%) were primary research articles (research involving the collection and analysis of primary data) and 60 (4.6%) were secondary research articles (research involving the analysis of secondary data). Among the primary research articles, there were 137 modelling studies and 108 survey studies. Of note, we identified five randomised controlled trials (RCT) carried out in Africa. In addition, we identified five systematic reviews including two meta-analyses.

The distribution of countries in which COVID-19 research was conducted is shown in table 1. Two hundred and sixty-three (20.3%) articles used the whole of Africa as their study setting while South Africa (15.4%), Nigeria (12.3%) and Ethiopia (6.8%) were the top three countries with the highest number of country-focused articles (table 1).

**Table 1:** Top 10 study settings in which COVID-19 articles were based on in Africa

Study setting	Number of studies (n=1296)	Non-original articles (n=630)	Primary research (n=606)	Secondary research (n=60)
Whole continent (Africa)	263	198	41	24
South Africa	200	104	89	7
Nigeria	160	56	98	6
Ethiopia	89	13	75	1
Egypt	70	15	54	1
Kenya	60	22	34	4
Uganda	51	22	29	0
Sub-Saharan Africa	48	34	12	2
Morocco	46	18	28	0
Ghana	45	20	25	0

### Research themes

Further, we classified all the articles into specific research groups to determine what the COVID-19 research trends are in Africa and to identify research gaps (table 2). Twenty-five per cent of the articles had a specific focus on assessing countries’ preparedness and response to the pandemic while 21.6% described the indirect health impacts associated with the pandemic.

**Table 2:** Overview of the top 15 topics covered in COVID-19 research articles in Africa

Study topic	Studies, n (%)
Country preparedness and response	323 (24.9)
Direct and indirect health impacts of pandemic	282 (21.6)
Transmission	181 (13.9)
Knowledge, attitudes and practice	117 (9.0)
Clinical characteristics	78 (6.0)
Socioeconomic impacts	73 (5.6)
Description of pandemic	47 (3.6)
COVID-19 testing	30 (2.3)
Genomic sequencing	19 (1.5)
Role of research	15 (1.2)
Clinical management	14 (1.1)
Ethics	14 (1.1)
Therapeutics and vaccines	13 (1.0)
Infection prevention and control	12 (0.9)
Impact of pandemic response	9 (0.7)
Other	69 (5.3)

Of note, only 1% of the articles focus on therapeutics and vaccines for COVID-19. The least represented research group is virus history and transmission (0.2%). These articles focus on the naming of COVID-19 and the implication of naming viruses by location and a review of the history of viral diseases in light of COVID-19.

### Authors

**Figure 2:**
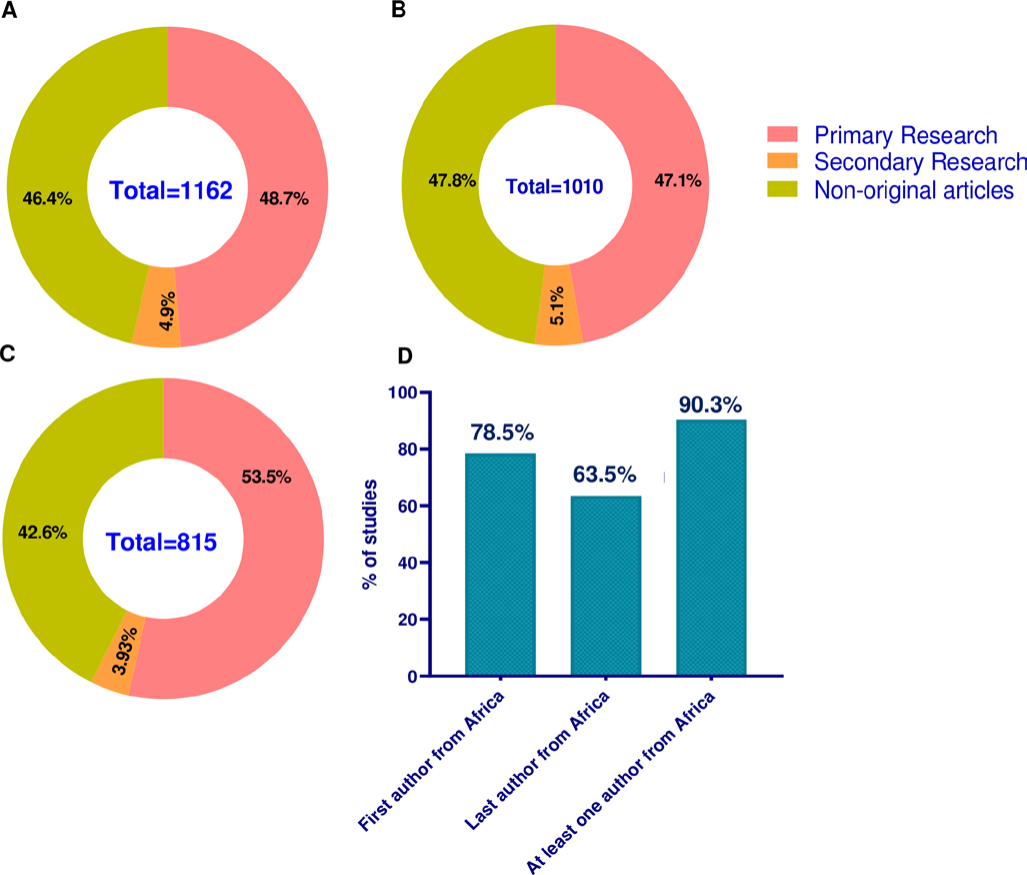
Proportion of articles authored by African researchers and the article type. (A) Proportion of research articles that have at least one African author. (B) Proportion of research articles that have an African first author. (C) Proportion of research articles that have an African last author. (D) Proportion of all articles by author contribution.

A total of 8669 authors were identified. Of this, 78.9% were affiliated with an African institution. 90.3% of these articles had at least one author affiliated to an African institution. 78.5% of all the articles reviewed had a first author affiliated to an African institution, while 63.5% had a last author affiliated to an African institution (figure 2D). There was an equal proportion of non-original (47.8%) and primary research articles (47.1%) published by African first authors (figure 2B). This was also the case for articles that listed at least one African author (figure 2A). On the other hand, a slightly higher proportion (53.5%) of the articles that listed an African last author were primary research articles (figure 2C).

### Author affiliation and network analysis

The top 15 institutions affiliated with the first and last authors are shown (table 3). Authors from the University of Cape Town (42) and Stellenbosch University (40) published the highest number of articles on COVID-19 in Africa. A network map of coauthors who contributed to COVID-19 research was created at the threshold of three documents per author, resulting in 147 collaborating authors (online supplemental figure 1).

**Table 3:** Top 15 institutional affiliations of first and last authors of published COVID-19 articles

Institutions	First authors (n)
University of Cape Town, Cape Town, South Africa	40
Stellenbosch University, Cape Town, South Africa	39
University of the Witwatersrand, South Africa	25
University of KwaZulu-Natal, South Africa	25
Makerere University, Uganda	22
University of Pretoria, South Africa	18
University of Lagos, Nigeria	14
University of Ibadan, Nigeria	13
University of Tripoli, Libya	13
London School of Hygiene & Tropical Medicine	12
KEMRI-Wellcome Trust Research Programme, Kenya	11
Groote Schuur Hospital, South Africa	9
University of Zimbabwe, Zimbabwe	9
Johns Hopkins Bloomberg School of Public Health, USA	9
Mohamed V University, Morocco	8

Institutions	Last authors (n)
University of Cape Town, South Africa	42
Stellenbosch University, South Africa	36
University of the Witwatersrand, South Africa	31
University of KwaZulu-Natal, South Africa	22
Makerere University, Uganda	22
University of Pretoria, South Africa	19
Cairo University, Egypt	13
University College London, UK	13
University of Ibadan, Nigeria	13
London School of Hygiene & Tropical Medicine, UK	13
University of Lagos, Nigeria	12
University of Oxford, UK	12
University of Tripoli, Libya	12
Groote Schuur Hospital, South Africa	8
KEMRI-Wellcome Trust Research Programme, Kenya	7

### Journals and funding

We ranked the top 15 journals that published the highest number of articles. One hundred and seventy-four articles (13.4%) had not yet been peer reviewed and were retrieved from the preprint server, medRxiv. Seventy-seven articles (5.9%) were published in the *Pan African Medical Journal* and 59 (4.5%) were published in the *South African Medical Journal* (table 4).

**Table 4:** Top 15 journals in which COVID-19 research in Africa was published

Journal	Studies (n)
medRxiv preprint	174
Pan African Medical Journal	77
South African Medical Journal	59
The American Journal of Tropical Medicine and Hygiene	31
International Journal of Infectious Diseases	27
Journal of Global Health	22
African Journal of Primary Health Care & Family Medicine	21
PLOS One	18
BMJ Global Health	15
Lancet Global Health	12
Clinical Infectious Diseases	12
BMJ	11
Lancet	11
Risk Management and Healthcare Policy	11
Travel Medicine and Infectious Disease	11

In addition, we ranked the top 15 funding bodies that provided funding for COVID-19 research in Africa (table 5). Of the studies that received funding and included information on the funding source, the Wellcome Trust (23 articles) and the National Institutes of Health (22 articles) were the top two funding bodies.

**Table 5:** Top 15 funding bodies that provided funding for COVID-19 articles in Africa

Funding body	Articles (n)	Country
Wellcome Trust	23	UK
National Institutes of Health	22	USA
Bill & Melinda Gates Foundation	20	USA
UK Department for International Development	12	UK
National Institute for Health Research	11	UK
South African Medical Research Council	10	South Africa
UK Medical Research Council	10	UK
European Union	9	EU
Global Challenges Research Fund	8	USA
USAID	8	USA
National Research Foundation	8	South Africa
DELTAS	5	Wellcome Trust and DFID Initiative
PEPFAR	4	USA
South African Department of Science and Innovation	4	South Africa
Canadian Institutes of Health Research	4	Canada

## Discussion

As the COVID-19 pandemic evolves in Africa and the rest of the world, efforts are being accelerated to identify effective preventive and therapeutic measures to mitigate its burden. Africa has contributed to this research and continues to build a body of evidence that can inform local interventions and mitigation strategies. This bibliometric analysis describes the COVID-19 research that has been carried out in Africa.

The analysis of the study designs published showed that almost half (48.6%) of the articles were commentary and editorial pieces and a similar proportion of publications were primary research articles (46.7%). Of note, we identified five RCTs carried out in Africa. RCTs are the gold standard for evaluating vaccines and treatment options for COVID-19 and require substantial skills and experiences. While it is encouraging that there are COVID-19 RCTs done in Africa, it is still a small figure compared with the number of other RCTs ongoing in the rest of the world.^17^ This presents a problem as findings from RCTs on different populations may not be generalisable to the African population. In addition, locally conducted RCTs are better placed to consider context-specific issues that would generate more effective interventions. More African countries must carry out or take part in RCTs to generate evidence that represents the African population. Secondary research articles represented only 4.6% of the included articles. However, this is expected as these types of articles require a large volume of primary research which is currently not available as COVID-19 is still a new research area.

Further, we explored the major themes in COVID-19 research in Africa. Most of the articles (25%) focused on describing the steps that African countries have taken to prepare and respond to the COVID-19 pandemic. This is expected as all countries took specific measures to prepare and respond to the pandemic. Research detailing these responses and their effects provides insight for further action and can inform future pandemics. Next, 21.6% of articles focused on the direct and indirect health impacts COVID-19 has had by highlighting the disruption and adaptation of health services catering to people with other diseases such as HIV and malaria. Articles describing transmission and clinical characteristics of the virus were another common field of study. Importantly, therapeutics and vaccine studies were under-represented (1%). This raises several concerns as control of COVID-19 will require safe and effective therapeutics and vaccines. First, a lack of local research informing vaccine and therapeutics development leads to an over-reliance on non-African countries to provide these interventions which will delay availability in Africa. Second, Africa requires interventions that fit the local context and is safe for the local population. Therefore, further research on prevention and treatment should be prioritised if we are to mitigate the effects of the pandemic.

Most of the articles included in the analysis focused on the whole continent as the study setting. In the remaining articles, South Africa was the dominant study setting identified. This finding is also in line with other studies that have assessed the volume of all health research from Africa where they report that South Africa has the highest publication output on the continent.^18–20^ This could be explained by several factors. For example, six of the top 15 institutions contributing to COVID-19 body of research in Africa are based in South Africa and increased capacity for research in terms of number of research institutions is important for increasing productivity. Nachega *et al* found that the number of public health research institutions present in an African country was associated with research productivity; for every additional research programme, public health research productivity increased by 241%.^21^ Second, in our analysis of institutions that fund COVID-19 research in Africa, we found that three of the top 15 funding bodies were from South Africa, with the other 12 being non-African. Availability of local funding increases publication output and is likely to focus on local issues. Finally, South Africa’s high gross domestic product (GDP) could also account for its high research productivity. Uthman *et al* reported that an independent factor that influenced research capacity was a country’s GDP.^19^ The lower number of articles from most African countries may be due to a lack of an enabling environment like that in South Africa. Progress towards controlling COVID-19 in Africa will require countries to sustain COVID-19 research and ensure that knowledge generated is also translated into effective policy. This means that African countries should strengthen local research capacity by increasing investment in research and supporting scientists on the continent.

Analysis on the authors of these articles indicates that 90.3% of the articles had at least one African author listed. While this proportion is high, it indicates that 125 articles (9.7%) in Africa and African countries lack African representation. Most (92) of these articles were editorial-type articles. Editorial-type articles are important as they provide insights into clinical care and health system responses. It was therefore encouraging to find that only a small proportion of these article types (14.6%) lacked African representation which is in contrast to previous findings.^22^ Non-Africa-based researchers often conduct research in Africa on behalf of external agencies in collaboration with African researchers. Although this may be important in the transfer of skills to African researchers, it can also lead to inequity in research partnership arrangements that put African researchers at a disadvantage. The representation and position of authors on publications can be used as a proxy to measure participation and leadership in research. Of the articles that had an African author, 78.5% listed an African first author and 63.5% had an African as last author. Previous studies found that African researchers were under-represented in studies on infectious diseases in Africa^23^; however, we find that in COVID-19 research, a high proportion of articles were by African authors. This could be due to the urgency of COVID-19 as a public health emergency which has led to a greater demand of research within the continent, increased funding, more time to write and publish and increased priority for COVID-19 publications.^24^ However, a limitation of this analysis is the use of institutional affiliation as an indicator of author nationality. This means that an African author based in a non-African institution would be classified as non-African, and a non-African working in an African institution would be classified as African. This misclassification underestimates the contribution of Africa’s large scientific diaspora. Additionally, there was disparity in the geographical representation of African first and last authors. The top four author affiliations were all South African institutions, with the University of Cape Town as the leading institution with the highest number of first and last authors. This is an indicator of the small number of research institutions on the continent and the uneven distribution of these institutions.

Over 13% of articles identified were published in the preprint servers. This is unsurprising as preprint articles represent a significant proportion of COVID-19 literature.^25^ However, for articles that were peer reviewed and published in journals, we found that three African journals featured in the top 15 journals publishing COVID-19 articles in Africa: *Pan African Medical Journal*, *South African Medical Journal* and *African Journal of Primary Health Care & Family Medicine*. This is promising as availability of local journals where African researchers can publish their work can increase publication output. Most of the research work that is undertaken in Africa is meant for the local audience and addresses local concerns and may not be fairly represented by western publishers. Therefore, it makes sense if such work are published in African journals. However, there is still a long way to go and the development of local journals and publishing houses should be encouraged so as to create a direct avenue for academics and researchers to publish their research findings.

In the articles that declared funding, most funding was obtained from organisations in the UK and USA. External funding has greatly contributed to increasing scientific research capacity on the continent. However, this may hinder development of sustainable African-led knowledge production and may deflect research priorities away from local needs. African organisations in the top 15 list of funding bodies were all from South Africa. Few studies were funded by African organisations, and specifically, African governments. This is similar to other previous studies which also observed few funders from the continent.^26 27^ Although increased investment in science by African governments is being made, the continent still falls behind other global regions with African countries spending less than 1% of GDP (on average) on research and experimental development.^28^

A major limitation of this study is the inclusion of only English articles. This means that we have missed out on a significant proportion of articles from African countries that do not use English as a main language. In addition, we did not critically appraise the studies/articles included in the analysis and therefore cannot assess the quality of research.

## Conclusion

The COVID-19 pandemic has highlighted the urgent need for research that informs effective action. Africa has contributed to this body of research despite the usual challenges associated with research and development in low-resource settings. Contrary to other studies on the output of research publication on infectious diseases, we find that African researchers have played a lead role in publishing COVID-19 research from the continent. However, this productivity is uneven across the continent with only a few countries accounting for majority of the publications. This points to the importance of support for research and development by African governments. In addition, more studies focusing on vaccines and therapeutics are needed to inform local development. Nonetheless, the effort to publish research by Africans for Africans is an indicator of the potential the continent has to contribute to global health research.

## Data Availability

Data are available upon request. All data relevant to the study are included in the article or uploaded as supplemental information.
